# BIOLOGICS: TARGET-SPECIFIC TREATMENT OF SYSTEMIC AND CUTANEOUS AUTOIMMUNE DISEASES

**DOI:** 10.4103/0019-5154.53175

**Published:** 2009

**Authors:** Siba P Raychaudhuri, Smriti K Raychaudhuri

**Affiliations:** *From the Department of Immunology and Rheumatology, VA Medical Center, Sacramento; Graduate Group Immunology, University of California-Davis, USA*

**Keywords:** *Biologics*, *anti-cytokines*, *TCR*, *B-Lymphocyte*, *immunonodulation*

## Abstract

Biologics are becoming important in the treatment of systemic and cutaneous autoimmune diseases. They are designed to target specific components of immune system. As the new drugs are capable of targeting proteins in a more specific fashion, yet have lower risks of systemic side-effects, they have considerable advantages over the older immunomodulators. The development of TNF-alpha blockers in the treatment of psoriasis, psoriatic arthritis, rheumatoid arthritis, Crohn's disease and ankylosing spondylitis have been major breakthroughs. Likewise, B-cell depletion has proved to be equally revolutionary for the treatment of lupus, pemphigus, certain vasculitides etc. But all said and done, the development of these molecules and their clinical usage are still at evolving stages. Consensus needs be formed to further categorize the clinical profiles of the patients in whom biologics are to be used in the future, given that the long-term safety profiles of these agents are very much unknown at present.

## Introduction

Immunomodulatory agents (biologics) are becoming important treatment alternatives for systemic and cutaneous auto-immune diseases. The Food and Drug Administration (FDA) considers a biologic to be any therapeutic serum, toxin, antitoxin, vaccine, virus, blood, blood component or derivative, allergenic product, analogous product, or derivatives applicable to the prevention, treatment, or cure of injuries or disease of man. Biologics for auto-immune diseases have been developed based on the current understanding of the abnormal immune system responses that contribute to the disease. Therefore, they are designed to target specific components of the immune system. The goal is to weaken or immobilize those features of the immune system that are triggering autoimmune diseases without the adverse side effects that can come from broadly weakening the immune system.[1] As the new drugs are capable of targeting disease-causing proteins in a more specific fashion, while also carrying lower risks of adverse effects, they have considerable advantages over traditional treatments. Biologics are having a major impact on the treatment of these diseases for which there has been significant unmet need for decades. Most of these agents are expensive, as is the laboratory monitoring for side effects that may be required. Cost-effectiveness may be a consideration in choosing to use a biologic agent and in picking among the available agents. An overview of biologic agents used in the management of patients with autoimmune disorders with a special emphasize on cutaneous diseases is presented here.

## Anti-cytokine Therapy

Cytokines play a pivotal role in the pathogenesis of inflammatory diseases. Cytokines are regulatory proteins produced by cells of the immune system and act as intercellular mediators in generation and control of immune and inflammatory response. In an inflammatory disease, several proinflammatory cytokines (such as TNF-alpha, IL-1, IL-6, IL-8, and IL-12) are counterbalanced by anti-inflammatory cytokines (such as IL-4, IL-10, IL-11, and IL-13).

The concept that specific types of immune responses are dominated exclusively by Th1 or Th2 profiles is now recognized as too simplistic to explain any rheumatic disease entirely. However, many biologic therapies have been developed for the purpose of targeting either the downregulation of proinflammatory Th1 responses or the upregulation of anti-inflammatory Th2 cytokine production. Thus, the Th1/Th2 paradigm is discussed briefly below.

### Th1 cells

Th1 lymphocytes participate in a broad variety of inflammatory responses, including cell-mediated inflammation in rheumatoid arthritis (RA), psoriasis, psoriatic arthritis, acute allograft rejection, graft-versus-host disease, and others. The list of proinflammatory mediators produced by Th1 cells includes but is not limited to the following:[2]

- Interleukin (IL)-2
- Interferon gamma
- TNF
- IL-12
- IL-15
- IL-18

### Th2 cells

Th2 lymphocytes stimulate antibody production by B cells and augment eosinophil responses. The activation of Th2 cells contributes to the development of chronic graft-versus-host disease, systemic lupus erythematosus, and systemic sclerosis. The list of mediators produced by Th2 cells includes but is not limited to the following:[2]

- IL-4
- IL-5
- IL-9
- IL-10
- IL-13

Since cytokines and their receptors are expressed outside the cell, they can be targeted by protein-based biologics like monoclonal antibodies and soluble receptor Ig fusion proteins and have become targets for drug development. In general, therapies designed to upregulate or augment the function of Th2 cytokines have been less successful in clinical trials than have interventions that target Th1 cytokine inhibition. To downregulate or inhibit the effector functions of cytokines *in vivo*, three general approaches have been used: (1) soluble receptor antagonists, (2) monoclonal antibodies to cytokines or their receptors, and (3) cell surface receptor antagonist proteins.

### TNF alpha blockers

Tumor necrosis factor (TNF; cachexin or cachectin) is a cytokine involved in systemic inflammation and is a member of a group of cytokines that all stimulate the acute phase reaction. TNF's primary role is in the regulation of immune cells. TNF is able to induce apoptotic cell death, inflammation, and inhibits tumorigenesis and viral replication. TNF is synthesized initially by activated macrophages and T cells as a transmembrane precursor protein. The cytoplasmic tail of this protein is then cleaved to release soluble TNF. The biological activity of TNF requires the aggregation of three TNF monomers to form trimeric TNF, which then acts by binding to one of two types of receptors: TNFR1 or TNFR2.[3,4] TNFR1 and TNFR2 are also known as p55 and p75, respectively. The trimeric structure of the receptors mimics that of the active cytokine.[5] TNF-R1 is constitutively expressed in most tissues, and can be fully activated by both the membrane-bound and soluble trimeric forms of TNF, while TNF-R2 is only found in cells of the immune system and respond to the membrane-bound form of the TNF homotrimer. As most information regarding TNF signaling is derived from TNF-R1, the role of TNF-R2 is likely underestimated. This binding causes a conformational change to occur in the receptor, leading to the dissociation of the inhibitory protein SODD from the intracellular death domain. This dissociation enables the adaptor protein TRADD to bind to the death domain, serving as a platform for intracellular signal transduction and activation of NF-kB and MAPK. These transcription factors then translocate to the nucleus and mediate the transcription of a vast array of proteins involved in cell survival and proliferation, inflammatory response, and anti-apoptotic factors. TNFR1 and TNFR2 both exert multiple effects on the immune system mediated inflammatory and proliferative cascades, including the following:[6]

- Stimulation of the release of the inflammatory cytokines interleukin (IL)-1beta, IL-6, IL-8, and GM-CSF.
- Upregulation of the expression of endothelial adhesion molecules (ICAM-1, VCAM-1, E-selectin) and chemokines (MCP-1, MIP-2, RANTES and MIP-1alpha)
- Direct effect on target tissues such as proliferation or apoptosis

Thus, TNF inhibitors offer a targeted strategy that contrasts with the nonspecific immunosuppressive agents traditionally used to treat most inflammatory diseases. There has been a major clinical breakthrough with the use of TNF alpha blocking biologics.[7] TNF alpha blockers are being used in a number of immunological diseases like psoriasis, psoriatic arthritis, Crohn's disease, RA, and ankylosing spondylitis [Table 1]. As of 2008, millions of patients have been treated with TNF alpha blockers for the treatment of inflammatory diseases.[8–12]

**Table 1:** FDA approved clinical indications for the use of TNF-α blockers

Rheumatologic indications
Severe and active RA, refractory to an adequate trial of disease modifying antirheumatic drugs (DMARDs)
Active polyarticular juvenile idiopathic arthritis (JIA), refractory to one or more DMARDs
Ankylosing spondylitis
Psoriatic arthritis
Gastrointestinal indications
Moderate to severe Crohn's disease (including fistulating Crohn's disease) with inadequate response to conventional therapies (TGA approved but not yet PBS listed)
Dermatological indications
Moderate to severe psoriasis

Three inhibitors of TNF are approved for the treatment of a variety of inflammatory illnesses by the United States FDA.

These medications are:

- Etanercept — a soluble p75 TNF-alpha receptor fusion protein
- Infliximab — a chimeric (mouse/human) anti-TNF-alpha antibody
- Adalimumab — a fully human monoclonal anti-TNF-alpha antibody

Etanercept (Enbrel) is administered by self-injection under the skin once or twice weekly. Marketed by Amgen and Wyeth, it received its first FDA approval in 1998. Etanercept is currently used to treat plaque psoriasis, psoriatic arthritis, ankylosing spondylitis, RA, and juvenile RA. A randomized trial of etanercept in 652 adult patients with active but stable plaque psoriasis involving at least 10% of the body surface area found three doses of subcutaneous etanercept (25 mg weekly, 25 mg twice weekly, 50 mg twice weekly) significantly superior to placebo.[13] Results of various clinical trials suggest one-half of the patients experienced a 75% reduction in psoriasis severity (PASI 75) after 12 weeks of twice weekly treatments.

Remicade (Infliximab) first received FDA approval in 1998 for the treatment of Crohn's Disease. It is marketed by Centocor. Subsequently, it received approval for use in patients with RA, ulcerative colitis, ankylosing spondylitis, psoriatic arthritis, and in 2006, severe plaque psoriasis. After the ASPIRE (active controlled study of patients receiving infliximab for treatment of RA of early onset) study, the FDA approved Infliximab to be used as first-line treatment with Mtx in moderate to severe RA. The study group patients receiving Infliximab+Mtx with early RA, less than 3 years were found to have less new joint erosions after 1 year than control patients. This emphasized the impact of early intervention in RA. Remicade is administered by intravenous infusion (IV) in a physician's office; receipt of a single dose takes 2 to 4 hours. Patients usually receive the first three doses within 10 weeks and then a dose every 8 weeks. Remicade is very effective for psoriasis. As an example, a multicenter randomized trial in 249 patients with severe plaque psoriasis found that compared with placebo, more patients treated with infliximab 3 mg/kg or 5mg/kg (given intravenously at weeks 0, 2, and 6) achieved at least a 75% improvement at week 10 (6% versus 72% and 88%, respectively).[14]

Adalimumab (Humira) first received FDA approval in 2002. It is currently used to treat psoriasis, psoriatic arthritis, RA, ankylosing spondylitis, and Crohn's disease. It is marketed by Abbott. Humira is very effective, with more than two-thirds of the patients in clinical trials experiencing a 75% reduction in psoriasis severity (PASI 75) after 16 weeks of treatment, including about 40% of patients who achieved a 90% reduction in psoriasis severity. Humira is administered by self-injection under the skin, typically once every 2weeks.

Etanercept is not a monoclonal antibody but a fusion protein that acts as a decoy receptor for TNF-α and acts competitively to inhibit the binding of TNF to its cell surface receptor; and also binds to the soluble form of TNF-α. Thus making TNF-α biologically inactive by inhibiting their interaction with the cell surface. All three agents block the biologic effects of TNF-α, although there are some differences in their structure, pharmacokinetics, and mechanisms of action. Both infliximab and adalimumab are anti-TNF-α monoclonal antibodies that bind specifically to human TNF-α with high affinity and neutralize the biological activity of TNF-α by inhibiting its binding to its receptors. The main difference between these two agents is that infliximab is a chimeric monoclonal antibody composed of both human and murine proteins and is given as an intravenous infusion; while adalimumab is entirely of human origin given as subcutaneous injections every 2 weeks. Methotrexate can be co-administered with infliximab to prevent the development of neutralizing antibodies to infliximab that could reduce its therapeutic efficacy. Adalimumab contains only human proteins so the chance of developing neutralizing antibodies is much less.

On the basis of experience gained in cytokine modulation therapy of chronic inflammatory diseases such as RA and psoriasis, the application of TNF- inhibitors represents a novel, more specific, and effective therapeutic option for distinct chronic inflammatory diseases. Various reports suggest that anti-TNF could be very effective in inflammatory skin diseases like Behçet's disease, pyoderma gangrenosum, cutaneous Crohn's disease, and subcorneal pustular dermatitis.[15]

Multiple adverse effects of TNF inhibition have been identified through both clinical trials and post-marketing surveillance.[16] The side effects of ant-TNF therapy are mentioned in Table 2. Although TNF-α blockers are generally well tolerated, physicians needs to be extremely cautious about the potential of serious side effects of anti-TNF drugs and should review the indications/contraindications of anti-TNF agents in every patient. The existence of any contraindications in the use of these agents [Table 3] needs to be considered before the commencement of therapy.

**Table 2:** Major adverse effects of anti-TNF therapy

Injection site reactions
Infusion reactions
Infections
Demyelinating disease
Heart failure
Malignancy
Induction of autoimmunity

**Table 3:** Contraindications for the use of TNF-α blockers

Absolute
Active infections (including infected prosthesis, severe sepsis)
History of recurrent or chronic infections (e.g., bronchiectasis)
After previous, untreated TB
Moderate to severe congestive cardiac failure
Multiple sclerosis or optic neuritis
Combination treatment with anakinra (IL-1 receptor antagonist)
Active or recent history (past 10 years) of malignancy except for skin cancer
Relative
Pregnancy
Lactation
HIV, hepatitis B, hepatitis C infection

A high incidence of latent tuberculosis (TB) is a major hurdle for the successful use of anti-TNF agents in Indian subcontinents. Re-activation of latent TB infection has been reported with the initiation of anti-TNF-α treatment; appropriate screening of patients with a Mantoux test and a chest X-ray should be performed before starting therapy. In PPD+ patients, it is preferable that a dermatologist works closely with a chest medicine specialist before prescribing any anti-TNF agent. Various uncommon infections such as listeriosis, disseminated histoplasmosis, and other deep fungal infections are reported among patients treated with anti-TNF agents. Dermatologists using anti-TNF should play an important role in educating their patients regarding the possible side effects of anti-TNF-α therapy and highlight some of the early warning symptoms. Patients should be instructed regarding the rudiments of differentiating simple viral illnesses and minor infections from those with the potential to cause serious harm, and should be instructed to inform their TNF-α inhibitor prescriber when signs of the more serious infections occur. Although rare, clinicians need to closely monitor for malignancy and induction of autoimmunity in patients receiving anti-TNF agents.

Conventional immunomodulatory agents (e.g., glucocorticoids, methotrexate, cyclophosphamide, azathioprine) are not without risks either. Thus, the decision to use an anti-TNF agent must be an individual one based upon the risk/benefit profile, severity of the disease, and involvement of the vital organs.

### IL-1 inhibitors

The original members of the IL-1 superfamily are IL-1α, IL-1β, and the IL-1 Receptor antagonist IL-1RA. The IL-1RA is a molecule that competes for receptor binding with IL-1α and IL-1β, blocking their role in immune activation. Both IL-1α and IL-1β are produced by macrophages, monocytes, and dendritic cells. They form an important part of the inflammatory response. IL-1 blockers are effective in animal models of RA but less effective than TNF alpha in human RA. A variety of approaches to IL-1 inhibition have been employed, which includes IL-1RA gene therapy, IL-1 trap, and Anakinra. The IL-1 trap comprises the extracellular domains of the IL-1 receptor accessory protein and the human IL-1 receptor, arranged inline and fused to the Fc portion of human IgG1. The clinical utility of cytokine traps is under investigation. Among these IL-1 inhibitors, Anakinra is only available for treatment. Anakinra (recombinant form of human IL-1 receptor antagonist) is approved in the USA for the treatment of moderate to severe RA with Mtx.[17] A combination of anti-TNF and anti-IL-1 therapy could be potentially dangerous. A study of the combination of Etanercept and Anakinra in patients treated unsuccessfully with Mtx showed no added benefit but an increase in serious infection (0% in Etanercept and 3.7-7.4% for combination therapy), injection site reaction, and neutropenia.[18] In comparison with the TNF inhibitors, IL-1 inhibitors have had a smaller impact on rheumatic disease.

### Other interleukin antagonists

IL-6 binds to both soluble and membrane-bound receptors and leads to the transduction of intracellular signals through the interaction of this complex with gp130, mediating gene activation and a wide range of biologic activities.[19] IL-6 has the ability to activate T cells, B cells, macrophages, and osteoclasts and is a pivotal mediator of the hepatic acute-phase response.

Tocilizumab (MRA) is a humanized anti IL-6 receptor antibody. Tocilizumab competes for both the membrane-bound and soluble forms of human IL-6 receptor, thereby inhibiting the binding of the native cytokine to its receptor and interfering with the cytokine's effects. It was tested in patients with RA and showed reduced disease activity and dose dependent improvement in ACR20.[20]

AMG714, a human monoclonal Ig G1 kappa anti IL15 Ab, has shown good response in RA.[21,22] In a Phase II study of patients with RA who had failed at least DMARD, 54% of the patients receiving 280 mg of AMG714 achieved ACR20 compared with 38% in the placebo group. A Phase I study of the new formulation of AMG714 is ongoing.

Interleukin (IL)-10 is a cytokine that has antiinflammatory and immunosuppressant properties. IL-10 plays a crucial role in several immune reactions, including regulatory mechanisms in the skin. In psoriasis, a common cutaneous immune disease, a relative deficiency in cutaneous IL-10 expression is observed. Several lines of evidence suggest that IL-10 could have anti psoriatic abilities. One pilot and two Phase II trials with sc IL-10 administration over 3-7 weeks in patients with moderate to severe psoriasis have supported this hypothesis.[23]

Interleukin 18 is involved in the Th1 immune response. Antibodies against interleukin 18 reduced the severity of colitis in animal models.[24] Clinical trials of a human anti IL-18 antibody or IL-18 binding protein are anticipated.[25]

## T Cell Targeted Therapies in the Treatment of Systemic and Cutaneous Autoimmune Diseases

Activation of T cells by antigen-presenting cells requires two distinct signals. First, the trimolecular complex must be formed, consisting of the T cell receptor (TcR), antigenic peptide, and MHC class II molecule from the antigen-presenting cell. The engagement of costimulatory receptors with its respective ligands provide an essential second signal for the optimal activation of T cells. A number of costimulatory molecules have been shown to influence T-cell activation. The most well-characterized T-cell costimulatory ligands are CD28 and cytotoxic T lymphocyte-associated antigen-4 (CTLA4) (CD152), which engage CD80 and CD86 receptors on antigen-presenting cells (APCs).[26,27] Among these, a principal signal is delivered by engagement of CD28 on T cells with CD80 (B7-1) and CD86 (B7-2) on APCs. This process enhances T-cell activation by stabilization of cytokine mRNA and upregulation of antiapoptotic genes. In contrast, CTLA4-Ig binds to B7-1 and B7-2 molecules on APCs and blocks the CD28-mediated costimulatory signal for T-cell activation. Thus, the B7 family of molecules on APCs regulates T-cell activation by delivering antigen-independent stimulatory signals through CD28 and inhibitory signals through CD152. This unique mechanism of T-cell activation has provided several target molecules for therapeutic manipulation of immune responses. These include:

- Inhibition of the second signal required for T cell activation: Targeting the various components of the T lymphocyte costimulatory systems such as CD28 (an activation receptor), CTLA4 (an inhibitory receptor) and CD80 or CD86 on antigen presenting cells. An alternative strategy could be to target other co-stimulatory molecules such as CD40/CD40 ligand system.
- Manipulation of T cell receptor (TcR) and MHC-antigen interactions: One approach is to identify the specific T cells that cause tissue injury and to produce monoclonal antibodies to the binding site of the T-cell receptor (TCR). A complementary strategy can be to identify the particular peptide sequence of the antigen molecule that is responsible for the initiation of the disease. A mimic of this peptide, referred to as an altered-peptide ligand can then be synthesized, which may block T-cell recognition at the level of the antigen-presenting cell. Attempts to prevent immune responses by targeting this approach remain unsuccessful because of the lack of information regarding the specific antigen(s) recognized by pathogenic T cells in autoimmune diseases.

The effectiveness of costimulatory signal blockade as a therapeutic device was shown over a decade by demonstrating that CTLA4-Ig inhibited graft rejection and induced long-term tolerance in mice.[28] Encouraging results in animal models have led to successful clinical trials with CTLA4-Ig in psoriasis and RA.[29,30]

To date, only costimulatory molecule inhibition with CTLA4-Ig (Abatacept) has been demonstrated to be effective in the treatment of RA and psoriasis. Abatacept (CTLA4-Ig) is a soluble fusion protein that consists of CTLA4 and the Fc portion of IgG1. Through its high affinity binding for CD28, CTLA4-Ig interferes with the binding of CD80 (B7-1) or CD86 (B7-2) to CD28, thereby inhibiting transmission of the second signal required for T cell activation. Abatacept should not be used concurrently with TNF inhibitors or with the interleukin-1 receptor antagonist anakinra because these combinations cause significant immuno-suppression and leads to severe infections. Live vaccines should not be given concurrently or within 3 months of stopping abatacept.

Currently we are working on an alternative approach to develop an immunomodulatory drug by manipulating CD28CD80/86 interactions using a monoclonal anti-CD28 antibody (FR255734) prepared by Fujisawa Pharmaceutical Co., Ltd. (now Astellas Pharmaceuticals Inc., Tokyo, Japan). FR255734 is a humanized IgG2κ anti-human CD28 antibody that has the complementary determining regions of the mouse anti-human monoclonal antibody TN228 and the Fc domain of human IgG2M3 in which two amino acid mutations (V234A, G237A) have been introduced into the human γ^2^ chain to eliminate binding of the antibody to FcγR. The original TN228 cell line was generated by immunizing BALB/c mice with human CD28-transfected mouse fibroblast L cells and fusing immune splenocytes with P3 U1 myeloma cells. The purified molecule consists of two heavy chains and two light chains, which are 447 amino-acid residues (C2177H3358N575O669S19; MW 48898.64) and 218 amino-acid residues (C1043H1628N279O342S7; MW 23772.21) in length, respectively. FR255734 binds to a human CD28-mouse IgG Fc fusion protein (*K*_d_ =3.72×10^−8^) and inhibits proliferation of human T cells stimulated with anti-CD3 and P815/human CD80^+^ cells in a concentration-dependent manner. FR255734 does not cross-react with mouse CD28.[31]

We have demonstrated by *in vitro* studies that FR255734 effectively inhibits cell activation by blocking CD28/B7 costimulatory interactions.[32] This encouraged us to evaluate the clinical efficacy of FR255734 in T-cell- mediated disease. To evaluate the therapeutic efficacy of FR255734 as a costimulatory antagonist, we have used the SCID mouse model of psoriasis. We noticed significant improvement in the thickness of the epidermis and reduction in infiltrates in the FR255734-treated group (*P*<0.005 at 10mg/kg and *P*=0.002 at 3mg/kg). In the normal saline-treated group and isotype controls (negative controls), epidermal thickness and the amount of infiltrates remained unchanged. The results of our study substantiate a novel approach for treatment of T-cell-mediated diseases by specifically manipulating the interaction of CD28 and B7 costimulatory molecules of activated T cells. It is expected that FR255734 will be effective in diseases associated with the active role of T cells such as psoriasis, RA, and multiple sclerosis.

### Targeting the effector memory T cells (T_EM_)

It has been reported that most T cells in psoriatic lesions are of the T_EM_ phenotype; thus, it would be desirable to selectively inhibit the function of these cells without affecting other T cells. LFA-3/IgG1 fusion protein (Alefacept^®^) preferentially targets T_EM_ cells and has been used for the treatment of psoriasis with partial success.[33] Alefacept binds to CD2, a receptor that is mostly expressed on T_EM_ cells and is critical for T cell activation. Alefacept is approved by the FDA for the treatment of adult patients with moderate to severe chronic plaque psoriasis who are candidates for systemic therapy or phototherapy. It is administered weekly for 12 weeks as a 15 mg intramuscular injection. CD4 cell counts should be checked every week or every other week while on therapy, and the dose should not be administered if the count is less than 250/μL. Alefacept should be discontinued if the CD4 count remains below 250/μL for one month. It is contraindicated in patients infected with HIV because of theoretical concerns related to the effects of Alefacept on CD4 cell counts.

### K^+^ channels in the immune system

K^+^ channels in humans are encoded by an extended superfamily of 78 genes and regulate membrane potential and Ca^2+^ signaling in both excitable and non excitable cells. Two of these channels, the voltage-gated Kv1.3 and the Ca^2+^-activated KCa3.1 channel are expressed in human lymphocytes where they play an important role in the T cell activation cascade. Engagement of the T cell receptor triggers a Ca^2+^-influx through voltage-independent Ca^2+^ channels, which results in an increase in cytosolic Ca^2+^ concentration necessary for the translocation of NFAT to the nucleus and the initiation of new transcription ultimately resulting in cytokine secretion and T cell proliferation. However, this crucial Ca^2+^-influx is only possible if the T cell can keep its membrane potential negative by a counterbalancing K^+^ efflux through Kv1.3 and/or KCa3.1. Both channels are therefore regarded as attractive new targets for immunotherapy: KCa3.1 for acute immune reactions mediated by naïve T cells and Kv1.3 for chronic immune reactions carried by memory T cells. We therefore believe that Kv1.3 blockers constitute a promising new drug candidate for the treatment of T_EM_-cell-mediated inflammatory skin diseases such as allergic contact dermatitis and psoriasis.[34] Because of their different mechanism of action, Kv1.3 blockers might also work in those patients with psoriasis who have no benefit from the existing therapies. For example, long-term therapeutic efficacy (PASI-75) of anti-TNF agents and Alefacept^®^ for psoriasis is only 60% and 20%, respectively and there currently still exists an urgent need for new psoriasis treatments. In addition, topical Kv1.3 blockers might be able to replace topical corticosteroids in patients with moderate psoriasis who are looking for a treatment with fewer side effects or a different side effect profile.

## B Cell Targeted Therapies in the Treatment of Systemic and Cutaneous Autoimmune Diseases

The major goal of B cell depletion therapy is to destroy malignant B lineage cells or autoimmune disease producing B cells in patients with cancers or autoimmune diseases, while at the same time retaining protective B cell immunity. For many years, rheumatologists have debated about how B cells contribute to the development of RA and whether depleting B cells in patients might be therapeutic. In a landmark study, Shlomchik, *et al.*[1] showed that autoimmune-prone MRL-lpr/lpr mice lacking B cells do not develop autoimmune kidney destruction, vasculitis, or autoantibodies.[35] They concluded that their “data demonstrate that B cells could be an important target for therapy of systemic autoimmunity” and that “elimination of B cells or B cell subsets would have distinct advantages over removal of Ig alone.” They turned out to be right. In a follow-up study by a different group it has been shown that MRL-lpr/lpr mice that have B cells but cannot make antibodies still develop autoimmune disease. This suggested that B cell depletion therapy might be able to work by removing B cell antigen presenting cells (APCs) presenting autoantigens as well by removing autoantibody producing B cells. Thus, B cells have a dual role in the pathogenesis of autoimmune diseases, which includes presentation of antigen to the T cells and antibody production. Similar to T cells, various markers of B cells such as CD20, CD22, and B cell growth factors like Blys and APRIL have been targeted to develop treatment for autoimmune diseases.

### B cell depleting agents

Rituximab — Rituximab is a B cell depleting monoclonal anti-CD20 antibody comprised of both mouse and human portions.

Ofatumumab — Clinical development is reportedly proceeding with fully human anti-CD20 monoclonal antibodies. One such agent, ofatumumab, is undergoing clinical trials to assess dosing, efficacy, and safety when used in patients with RA.

Belimumab — Belimumab is an anti-BLyS monoclonal antibody (LymphoStat-B) that has been used in a dose ranging, Phase II trial in RA and lupus.

Atacicept — Atacicept is a recombinant fusion protein comprised of a portion of the transmembrane activator and calcium-modulator and an immunoglobulin chain (TACI-Ig or atacicept). Atacicept targets molecules on the B cell surface that promote B cell survival (BLyS and APRIL).

Among all these B cell depleting agents, only Rituximab is currently in clinical use. Rituximab is a chimeric monoclonal anti-CD20 antibody that selectively depletes CD20-expressing B cells. It has been used extensively to treat non-Hodgkin's lymphoma and it has also received approval in the United States and Europe to treat RA unresponsive to a TNF blocker.[36]

Data suggest that for patients with severe SLE who have failed to respond to conventional treatment, the combination of Rituximab and cyclophosphamide can provide a new therapeutic alternative.[37] There are various other specific uses of Rituximab for cutaneous diseases that are currently in the developing stage. Rituximab is highly effective in pemphigus.[38] The dose of Rituximab (unlabeled use) for pemphigus vulgaris has been proposed as 375 mg/m^2^ once weekly for weeks 1, 2, and 3 of a 4-week cycle, repeat for 1 additional cycle, then 1 dose per month for 4 months (a total of 10 doses in 6 months). Rituximab appears to be of benefit in patients with ANCA positive vasculitis. Rituximab has also been found to be effective in complicated dermatomyositis.

### Angiogenesis factor

Angiogenesis plays an integral role in psoriasis and RA by supplying oxygen and nutrients necessary for cell metabolism and division, as well as by bringing in leukocytes and signaling mediators such as cytokines, chemoattractants, and growth factors. As the synovium/epidermis expands, more blood vessels are needed to supply poorly perfused and oxygenated areas distant from the pre-existing blood vessels. This promotes formation of further blood vessels (angiogenesis). A range of different factors can promote angiogenesis, including fibroblast growth factor (FGF)-1 and FGF-2, angiopoietins, and vascular endothelial growth factor (VEGF). VEGF inhibition has been shown to be effective in models of arthritis, including CIA.[39,40] VEGF inhibition *in vivo* is, however, associated with side-effects such as impaired wound healing, hemorrhage, and gastrointestinal perforation. As a consequence, other members of this family have been targeted. Placental growth factor (PlGF), such as VEGF, binds to VEGF-R1 (and soluble VEGF-R1), but, in contrast to VEGF, PlGF does not bind VEGF-R2.[41,42] PlGF appears not only to induce distinct signaling events via VEGF-R1, but also to amplify VEGF-driven effects through VEGF-R2 and to complex with VEGF/VEGFR2 forming heterodimeric complexes that transphosphorylate each other.[43] Interestingly, PlGF-deficient mice are fertile, viable, and do not display major vascular abnormalities.[44] Instead, PlGF may play a more pronounced role in pathological angiogenesis, as shown by impaired tumor growth and vascularization in mice lacking this molecule. Furthermore, PIGF is expressed in synovial fluid, making it a potentially important therapeutic target.[45]

### Drugs that inhibit leukocyte adhesion

Blockage of leukocyte migration has been proposed as a means of downregulating inflammation. Intercellular adhesion molecule-1 (ICAM-1) is a transmembrane glycoprotein that has multiple functions involving propagation of inflammatory processes, and is upregulated in inflammatory bowel disease. Lymphocyte function-associated antigen 1 (CD11a) mediates interactions between T cells and mononuclear phagocytes through its ligand, the ICAM-1 (CD54).

Efalizumab — Multicenter randomized, controlled trials have shown that efalizumab (Raptiva), a humanized monoclonal antibody to CD11A, has benefit in the treatment of psoriasis.[46]

As an example, a randomized trial found that subcutaneous efalizumab (1 or 2 mg/kg/week) was significantly superior to placebo. After 12 weeks, there was at least a 75% improvement in a psoriasis severity index in 22, 28, and 5%, respectively. Among patients who initially improved at least 75% after 12 weeks of efalizumab, improvement was maintained through 24 weeks in 77% of those who were randomly assigned to continue efalizumab and in 20% of those who switched to placebo, and more patients with lesser degrees of initial improvement showed continued improvement with efalizumab than with placebo. Adverse events including headache, chills, pain, and fever were more common in patients receiving efalizumab, but serious adverse events and infections were no more common than in those receiving placebo.

Efalizumab is approved by the FDA for adults with chronic moderate to severe plaque psoriasis; it is administered weekly via subcutaneous injection with a recommended initial conditioning dose of 0.7mg/kg followed by weekly doses of 1mg/kg (maximum single dose of 200mg). It is recommended that platelet counts be monitored periodically in patients treated with efalizumab as there have been reports of thrombocytopenia. There have also been case reports of hemolytic anemia and severe infections in patients receiving efalizumab.

## New Generations of Biologics

Golimumab (CNTO M8) is a fully human anti-TNF alpha mAb; it is a newer TNF alpha that is being studied. In a double-blind, placebo-controlled, dose ranging Phase II study of 172 adults with RA more than 3 months and refractory to Mtx, 79% of the patients who received 100 mg of Golimumab every 2 weeks with Mtx showed an ACR20 response at 16 weeks compared with 37.1% in the placebo (Mtx alone) group. A total of 75% of the patients treated with Golimumab and Mtx experienced 20% improvement in arthritic symptoms (ACR20) at 52 weeks.[47] Its use in psoriatic arthritis and ankylosing spondylitis is in trial. Humanized PEGylated Fab fragment antitumor necrosis factor-alpha mAb developed by Celltech/UCB has shown promise in the treatment of Crohn's disease and is less immunogenic than conventional mAbs.

Another approach is to target IL23 or IL-6, which is necessary for differentiation and survival of Th17. IL23 deficient mice are found to be resistant to experimental autoimmune encephalitis, CIA, and inflammatory bowel disease.[48–50] Th17 cells express ROR gamma transcription factor and IL17A and IL17F. IL17 induces TNF alpha and IL-6, growth factor (GM-CSF and G-CSF), and chemokines CXCL8, CXCL1, and CXCL10. Blockade of Th17 has been shown to be effective in a number of animal models of disease including CIA,[51–53] hence it is a target for psoriasis and RA.

IL-23 induces IL-22 in the Th-17 cells. In RA, both IL-22 and its receptor IL22R1 are expressed in synovial tissues and r IL-22 was shown to increase MCP-1 expression and proliferation of fibroblast *in vitro* suggesting a pro-inflammatory role. In Crohn's disease, it has a protective role by upregulating LPS binding protein thereby reducing LPS. Further work needs to be done to find its role in other autoimmune diseases.

The recognition that nerve growth factor (NGF) and its receptor system (NGF-R) has a critical role in pathomechanisms of inflammation, inflammatory disease, and pain mechanisms has provided unexpected and attractive opportunities for developing a novel class of therapeutics for inflammatory diseases and chronic pain syndromes.[54] We have demonstrated that K252a, a high affinity receptor inhibitor and neutralizing NGF antibody are therapeutically effective in the treatment of psoriasis.[55] Several investigators and pharmaceutical companies are currently in search of anti-NGF therapy for inflammatory diseases, arthritis, and pain control. Pincelli and his colleagues have extended our observations and are currently in the process of preparing a topical preparation of K252a for the treatment of psoriasis.[56] Recently, Shelton, *et al.* from Rinat Neuroscience Corp have reported that treatment with anti-NGF antibody is efficacious for autoimmune arthritis in rats.[57] These results encouraged Rinat to extend their study in chronic painful human diseases such as osteoarthritis (OA).[58]

## Conclusion

Since the late 1990s, the success of biologic agents in the treatment of RA has dramatically altered the approach to treating this disease and a variety of other inflammatory illnesses. The development of TNF alpha blocker biologics for the treatment of psoriasis, psoriatic arthritis, RA, Crohn's disease, and ankylosing spondylitis is a major breakthrough in the treatment for autoimmune diseases. On the other side, B cell depletion therapy has provided a very effective therapeutic option for critical patients of lupus, pemphigus, RA, and ANCA-associated vasculitis. Biological treatments are relatively expensive and, given the widespread patient dissatisfaction with conventional therapy, the demand for them is high. The clinical experience of biological therapies in dermatology is currently an ongoing process and the long-term safety is uncertain. There is a need to better define which patients should be considered for biological therapy. High cost and the potential for serious side effects of biologics are social and clinical challenges to the present generation of dermatologists.
